# T2D-Db: An integrated platform to study the molecular basis of Type 2 diabetes

**DOI:** 10.1186/1471-2164-9-320

**Published:** 2008-07-07

**Authors:** Shipra Agrawal, Nevenka Dimitrova, Prasanthi Nathan, K Udayakumar, S Sai Lakshmi, S Sriram, N Manjusha, Urmi Sengupta

**Affiliations:** 1 Institute of Bioinformatics and Applied Biotechnology, Bangalore, India; 2 Philips Research Asia, Philips Innovation Campus, Manyata Tech Park, Nagawara, Bangalore, India

## Abstract

**Background:**

Type 2 Diabetes Mellitus (T2DM) is a non insulin dependent, complex trait disease that develops due to genetic predisposition and environmental factors. The advanced stage in type 2 diabetes mellitus leads to several micro and macro vascular complications like nephropathy, neuropathy, retinopathy, heart related problems etc. Studies performed on the genetics, biochemistry and molecular biology of this disease to understand the pathophysiology of type 2 diabetes mellitus has led to the generation of a surfeit of data on candidate genes and related aspects. The research is highly progressive towards defining the exact etiology of this disease.

**Results:**

T2D-Db (Type 2 diabetes Database) is a comprehensive web resource, which provides integrated and curated information on almost all known molecular components involved in the pathogenesis of type 2 diabetes mellitus in the three widely studied mammals namely human, mouse and rat. Information on candidate genes, SNPs (Single Nucleotide Polymorphism) in candidate genes or candidate regions, genome wide association studies (GWA), tissue specific gene expression patterns, EST (Expressed Sequence Tag) data, expression information from microarray data, pathways, protein-protein interactions and disease associated risk factors or complications have been structured in this on line resource.

**Conclusion:**

Information available in T2D-Db provides an integrated platform for the better molecular level understanding of type 2 diabetes mellitus and its pathogenesis. Importantly, the resource facilitates graphical presentation of the gene/genome wide map of SNP markers and protein-protein interaction networks, besides providing the heat map diagram of the selected gene(s) in an organism across microarray expression experiments from either single or multiple studies. These features aid to the data interpretation in an integrative way. T2D-Db is to our knowledge the first publicly available resource that can cater to the needs of researchers working on different aspects of type 2 diabetes mellitus.

## Background

T2DM previously known as non-insulin dependent diabetes mellitus (NIDDM) is a multifactorial and genetically heterogeneous disease caused by impairment in both insulin secretion and insulin action [1-5]. A total of 180 million people are affected and the prevalence of T2DM is continuously rising throughout the world. The number affected is estimated to be more than double by 2030 [6]. The primary characteristics of this disease include insulin resistance, relative insulin deficiency and hyperglycemia. The disease develops due to genetic predisposition and environmental factors such as improper food-habits, obesity, sedentary lifestyle etc. Insulin deficiency is a consequence of hyperglycemia, it causes impaired insulin-mediated glucose uptake from muscle, increased hepatic glucose production and also an increase in the mobilization of free fatty acids from adipose tissue, decreased mitochondrial biogenesis, and reduced ATP biosynthesis etc. [7-10]. Studies indicate that insulin resistance alone does not result in T2DM unless there is an β-cell inability to compensate for the insulin resistance with appropriate hyperinsulinemia [9,11]. β-cell dysfunction is characterized by the impairment of insulin secretion [2,12]. Increased levels of non-esterified fatty acid (NEFA), glycerol, Tumor Necrosis Factor-α (TNF-α), cytokines in the blood plasma and the other environmental and genetic risk factors adversely affect the insulin signaling cascade [13-15]. A reduced level of adiponectin, an insulin sensitizing hormone from adipocytes, is also a predictive signal for this disease [16,17]. Obesity is associated with insulin resistance and it is reported to be a major risk factor responsible for the development of T2DM [15,18].

In terms of correlating genetic predisposition and T2DM, multiple chromosomal regions have been reported to harbor susceptibility genes [3,5,19]. Some common gene variants, reported to be consistently associated with T2DM include those of calpain 10 (CAPN10) [20], PPAR-γ coactivator 1 (PGC1) [21], Pro12Ala PPAR-γ (PPARG) [22], Glu23Lys potassium inward rectifying channel (KCNJ11) [23], Hepatocyte nuclear factor-4 alpha (HNF4α) [24,25], the glucose transporter (GLUT2) [26], transcription factor 7-like 2 gene (TCF7L2) [27] and retinol binding protein (RBP4) [28].

The progress of research on etiology, genetic predisposition, role of environmental and other risk factors, characterization of candidate genes and other molecular, genetics and biochemical parameters has led to the generation of voluminous data. Hence, availability of all datasets as an integrated resource would be useful in acquiring a better understanding of pathophysiology of the disease. Considering the impact of such information in facilitating diabetes research and drug design, and with the aim of providing easy access to the large and increasing volume of data, we have developed a comprehensive web resource on T2DM and named it as T2D-Db. This compliments another publicly available resource on Type 1 diabetes (T1Dbase) on diabetes research [29].

T2D-Db provides an user-friendly platform with exhaustive search options for the retrieval of all reported information on candidate genes in human, mouse and rat. The database has been organised into candidate genes, gene expression data, pathways, protein-protein interaction, SNP marker data and risk factors/complications. It facilitates a graphical as well as a tabular display of SNP markers in a gene/genome-wide map for a user selected chromosomal segment in a selected organism. Furthermore, T2D-Db provides tabular and heat map presentation [30] of the microarray expression data of selected gene(s) in an organism across multiple experiments. Network visualization and analysis for interaction data has also been made possible through Cytoscape web-launch [31]. Every module of the database has been linked with other relevant external resources and the literature for further information on the selected component. The database will be updated periodically with enhanced features and new candidate genes as and when they are reported. We also urge the diabetes research community to submit data to keep this public platform updated. Finally, T2D-Db with the aforesaid features will be an extremely useful resource in analyzing and correlating the available research data on the molecular basis of type 2 diabetes.

### Motivation for T2D-Db

The development of T2D-Db has been motivated by the intense impact of this disease on human health. The ever-increasing complications of T2DM have led researchers to do intensive studies on this disease. Continuous research efforts in this field have led to the accumulation of huge amount of data. Further research on this complex and multifactor/polygenic type 2 diabetes requires collation of all the known candidate genes and related information to understand the complete molecular pathophysiology of the disease. Though, the candidate gene/region and other molecular data is available through different databases, it is very inconvenient to study the diasese by searching the scattered information. The entire information has been integrated in T2D-Db. The user can search and obtain the data as a desired table/graphical display quickly and efficiently from a single resource. The data organization and tool features provide much more meaning to the data than the data available from individual resources. An integrated comparison and analysis of the candidate genes will help a researcher to assess the importance of these genes in the disease that might further give an insight for designing the newer and powerful drugs against this disease.

The user-friendly and freely accessible T2D-Db is expected to serve the research community with a huge amount of information to accelerate the progress of work in this field.

## Database implementation

### Database contents

The candidate gene data has been manually curated from the Pubmed literature. We could extract 159, 59, and 36 empirically defined candidate genes for human, mouse and rat respectively. The annotation based searches for these reported genes were made from NCBI Entrez gene database [32]. Supplementary data for each candidate gene were also taken from OMIM [33], MGI [34], RGD [35], and Ensembl [36] databases. The nucleotide sequence of each candidate gene was downloaded from NCBI nucleotide database. The following criteria have been used to search the candidate genes from Medline literature:

i. PubMed and GoPubMed databases have been searched thoroughly giving different keywords related to the disease like: Type 2 diabetes, non-insulin dependent diabetes mellitus and insulin resistance etc.

ii. After the careful extensive reading of the research article, we identify the candidate genes for diabetes and associated risk factors and complications. The data is further validated twice by senior curators and then included in T2D-Db.

iii. OMIIM (for human gene), MGI (for mouse gene), RGD (for rat gene) and Ensembl databases have been used to cross validate the function of each gene gene.

Gene orthologs of each candidate gene for each organism were searched and collected from NCBI Entrez gene database (Human-NCBI 36.2, Mouse-NCBI M37.1, Rat-RGSC v3.4) [32]. Information on putative homologs of candidate genes in different organisms, and transcript sequences from the same transcription locus were collected from NCBI Homologene (Release 61) [37] and Unigene databases (Build#210) [38] respectively. Transcript information of candidate genes was taken from the database of transcripts (DoTS) of EPConDb (version 4.1) [39].

In addition to gene related information, we included gene ontology based information such as biological function, process and molecular component of candidate genes from Gene Ontology database, Amigo (Release 2008-04-10) [40]. In order to be more comprehensive, data under other important categories have also been included. For instance, information regarding biological pathways involving candidate genes was retrieved from KEGG (Release 43.0) [41], Biocarta [42] and Reactome (Version 22) [43] databases. Protein-protein interaction data for each candidate gene of human, mouse and rat was collected from the APID (Release July 2007) [44] and PIP [45] databases. The SNP markers corresponding to each susceptible gene and chromosomal segments have been downloaded from dbSNP (Build 126) [46]. EST information was extracted from dbEST (Release 041108) [47]. Tissue specific gene expression data was downloaded from GNF SymAtlas (Version 1.2.4) [48]. The normalized microarray expression data has been taken from NCBI Gene Expression Omnibus (GEO database) [49] and relational tables were generated for all the experimental data.

The risk factor/complications data is collected as follows: The information on factors increasing the risk/complications of this disease has been collected through the Medline literature search. Following this, each risk factor is searched thoroughly for its association with T2D and the responsible candidate genes have been collected, validated and organized in T2D-Db.

### Value addition and data integration in T2D-Db

The candidate gene/candidate region data has been collected and annotated with all reported molecular information on these genes and systematically organized in T2D-Db. Besides, the seamless organization of molecular data for each candidate gene, this platform also facilitates an online systems biology analysis of the selected candidate genes and their interacting partners. Simultaneously, it caters genome wide map display of SNP, Genome Wide Association (GWA) and EST data along with the information on associated genes in a selected genomic stretch. One can also visualize the expression patterns of candidate genes and other important genes from microarray expression datasets through an expression viewer. It also supports information on risk factors and complications of type 2 diabetes. T2D-Db will support the continuous need of updated information on above mentioned molecular data and other important facts associated with this disease.

### Database design and organization

The web interface of T2D-Db has been developed using HTML, DHTML and Javascript. All data in the database have been stored in MySQL tables. CGI-Perl has been used as an interface layer between the front and back ends of T2D-Db. CGI-Perl scripts process all the queries from the user by fetching the data from the database and displaying it on the webpage after proper formatting of the results. FASTA formatted nucleotide sequences of genes have been stored in flat files, which can be downloaded as well. Additional file 1 describes the data flow through the database application. The database has been integrated with the following three tools to facilitate the meaningful graphical representation of the data.

#### 1) Microarray expression viewer

The Microarray Expression Viewer is a Java based application, created to provide a visual display of gene expression data for a set of selected genes across a set of selected experimental studies. Java Web Start is used for launching the application on the user's machine. The .jnlp file for launching the application is created dynamically upon querying the search option 'heat map-view'. All the studies, experiments and the corresponding gene expression values are collected into a text file, which is an input to the viewer application. The heat map generated can be explained as follows:

(i) The Heat Map shows the labelled samples (both controls and diseased ones) and the expression of genes. (ii) It displays the intensity level for each gene as red, green or yellow spot. A red spot indicates increased expression of a particular gene in the diseased samples. A green spot indicates normal expression and a yellow spot indicates no change in the expression levels between the controls and the diseased samples.

#### 2) Protein-protein interaction map

Cytoscape 2.5.1 [31], a publicly available Java-based molecular interaction network visualization tool has been integrated with T2D-Db to customize and facilitate protein-protein interaction network analysis. The network for a selected gene will be generated and launched on Cytoscape panel on the user's machine using the Java web start technology. The data sources for network analysis have been procured from APID and PIP databases. The user can select either of the two data sources to view the interaction network for a particular gene.

#### 3) SNP Marker map

The 'SNP map' generates a dynamic map displaying SNP markers and the corresponding genes in selected chromosomal regions of specific chromosomes in any of the three organisms. The map shows the names of the genes and SNPs on mouse over. Names are further externally linked to Entrez gene database and dbSNP respectively. MySQL tables have been created for both SNP marker and gene data, which in turn are relationally linked to each other. Perl programs using the GD module generate the map view and allow the user to view SNP markers in a selected chromosomal segment on the map. Besides the display of candidate gene specific SNP markers from dbSNP, SNP map displays both tables and graphical view of all T2D associated markers from dbSNP and Welcome Trust Case Control Consortium (WTCCC) in a selected human genomic stretch. The SNP markers have been provided with the external links to the HapMap database (50).

#### 4) EST Marker map

EST information has been provided for a better understanding on the expression behavior of particular candidate genes. T2D-Db provides an EST map tool that maps and displays ESTs and the associated genes in the selected regions on specific chromosomes. These data is further externally linked to NCBI dbEST and Entrez gene databases respectively.

#### 5) T2D Gemone-wide association studies

Genome wide association study shows the genetic variations associated with a particular disease. The GWA studies on diabetic vs control subjects have been collected from WTCCC (51). The data is displayed as tables and map at a genome wide scale. T2D-Db also provides an external link to each candidate gene in Genetic Association Database (GAD).

## Results and Discussion

**Figure 1:**
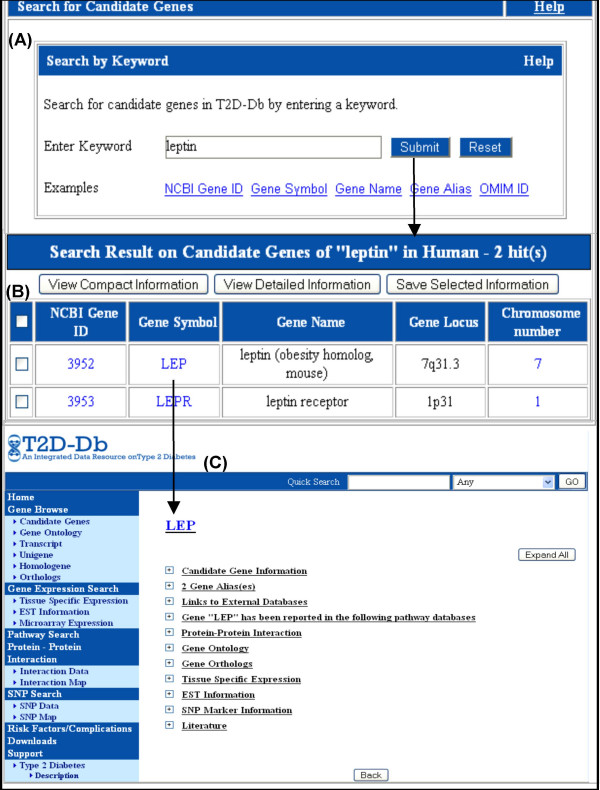
**Querying a candidate gene through simple search option (A-C)**. (A) Querying a candidate gene search page by using gene name as keyword. (B) Candidate gene result page for the keyword 'leptin'. Options are provided to view the compact and detailed information for the selected genes in this page. (C) The detailed gene information page, which is obtained by clicking on 'Gene symbol' in the previous page. This page provides all associated information for the selected gene.

**Figure 2:**
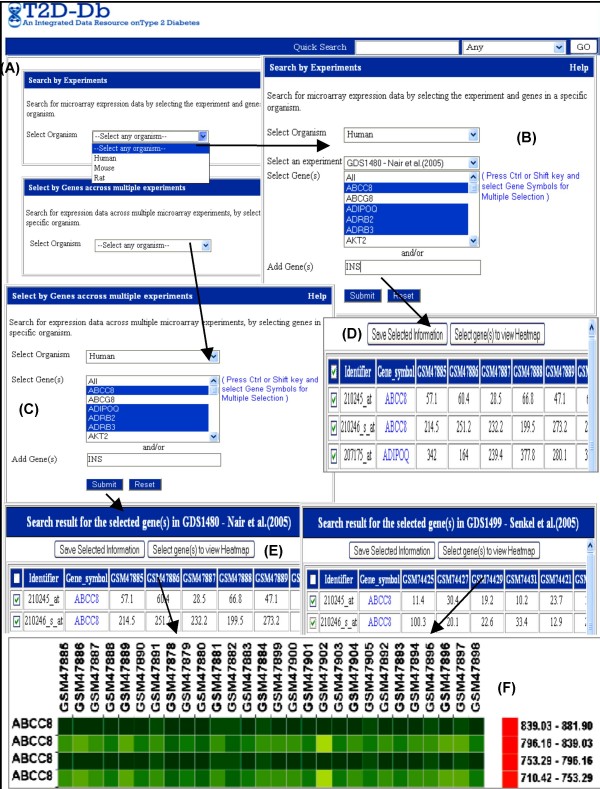
**Screenshot of search results from a sample query showing microarray expression data for selected genes in T2D-Db (A-F)**. (A) Search page for entering the gene and/or organism name. (B) In the 'Search by Experiments' option, if the user selects an organism, the microarray experiment along with the corresponding genes present in the candidate gene list will be displayed. The user can also specify other genes in 'Add Gene(s)' section. (C) The user can search for patterns of gene expression present in multiple studies by using the 'Select by Genes across multiple experiments' option. Here on selecting the organism same kind of gene list will be displayed. (D) & (E) After submitting the query as mentioned in (B) and (C) output pages will be displayed, which contain the gene symbol, corresponding probe id and expression values across different samples for the corresponding selected genes. (F) Selection of desired gene entries and clicking on "Select gene(s) to view Heatmap" facilitates visualization of the heatmap to represent the expression pattern graphically. The red, green and yellow colours describe increased, no change and decreased expression respectively.

**Figure 3:**
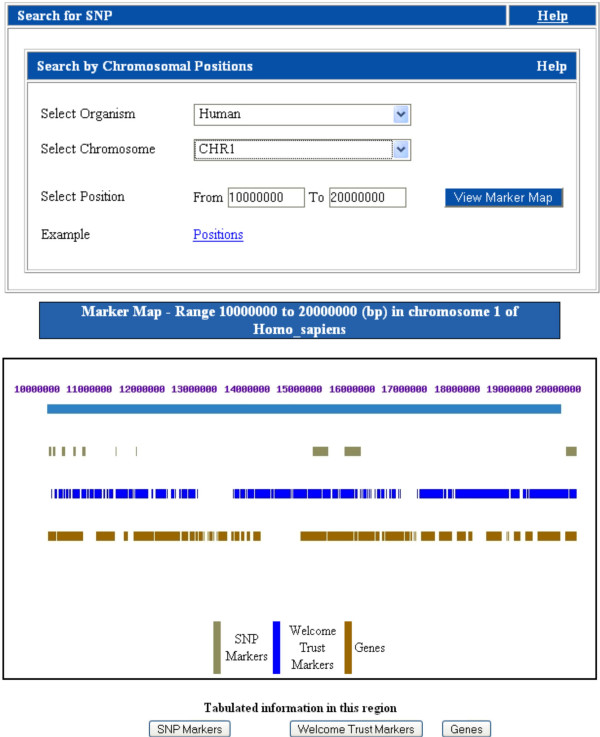
**Screenshot of a SNP marker search result page and SNP map**. An output page of SNP marker search for chromosomal region (10000000–20000000) of human chromosome 1. The 'View Marker Map', shows the labelled and dynamic map of SNP markers and the corresponding genes in the selected chromosomal segment. Tabulated information is also available for all SNP markers and Genes found in that selected region. The chromosomal segment, SNP markers and genes in the map have external links to the UCSC browser, dbSNP and NCBI Entrez gene respectively.

**Figure 4:**
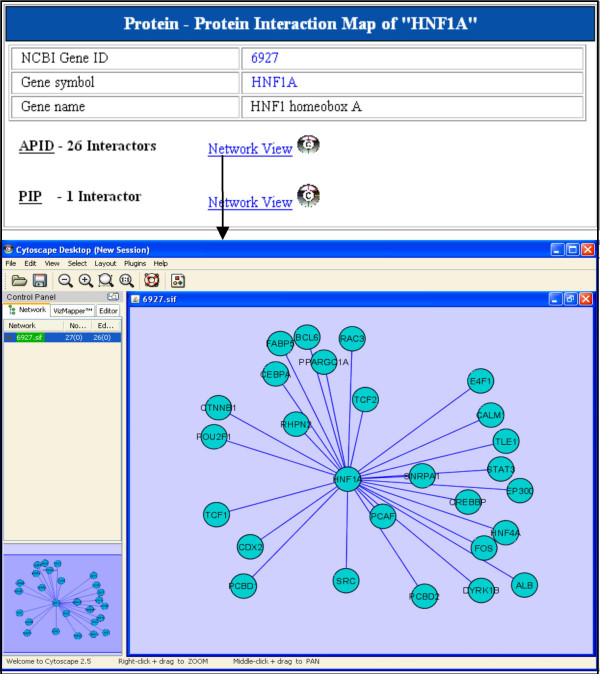
**The protein-protein interaction map**. The results page shows the protein-protein interaction map for the gene HNF1A. The interaction data from APID and PIP databases can be visualized separately. Clicking of the icon 'Network view' of either of the data sources (APID or PIP), the interaction map for the corresponding genes will appear on the cytoscape panel.

All information related to the reported candidate genes of T2DM is available in T2D-Db. A variety of search options have been created, which provide easy and quick access to all the modules of the database. The search options are self explanatory and highly specific for each section of the database. Some important features of the database are listed below:

a) T2D-Db provides both compact and detailed information on each candidate gene. The compact view section gives a precise description about the gene name, gene locus, and gene ID and gene symbol hyperlinked with NCBI Gene and T2D-Db Gene page respectively. The detailed view provides gene summary, OMIM ID and Ensembl ID with external links to their respective databases and literature sources. The gene summary includes gene name, gene symbol, gene aliases, a precise description, gene locus, chromosomal number and the related literature. It also includes data under several different categories, which can be expanded further to view or save in detail (Figure 1). Candidate genes can be searched through key words, organism selection and also browsed alphabetically for human, mouse and rat. There are other supplementary search modules on candidate gene i.e, Gene ontology, Orthologs, Homologene, Unigene, Transcripts and so on. Figure 1(A-C) exemplifies the schematic flow of information when a query on specific candidate gene is submitted.

b) A quick search option has been provided to ease access to all the information available on candidate genes of human, mouse or rat. The user can enter any query and select the relevant information such as Candidate genes, Markers, Pathways and Risk factors/complications from a dropdown menu to search the database.

c) The gene symbol in every section has a link to a T2D-Db result page, which contains exhaustive data for the selected gene such as candidate gene information, gene aliases, orthologs, pathways, protein-protein interaction data, gene ontology, SNP markers, gene expression data, literature and links to the respective external databases.

d) The Gene Expression Search section provides tissue specific gene expression data, and all the available EST information for each gene with external links to NCBI dbEST. Additionally, expression values for each gene from single or multiple microarray datasets can be curated through this database. The microarray expression data search option provides an opportunity to the user to select a set of specific experiments, and analyze and visualize the results comparatively to formulate their own hypothesis. The gene expression pattern of a set of user selected genes across single or multiple experiments in an organism can also be visualized as a heat map. In addition to the selection of candidate genes, the user can add other gene(s) of interest to obtain a collective view of the expression pattern. Figure 2 describes the flow of query processing to extract microarray expression data of candidate gene(s) across single or multiple experiments in Human as an illustration.

e) Pathway information on candidate genes participating in different biological pathways is provided with their names and IDs from Biocarta, KEGG and Reactome databases.

f) SNP markers found in a specific candidate region of a chromosome in a selected organism can be viewed as an 'SNP map'. This marker map displays the names, chromosomal localization of genes and corresponding SNP markers in a chromosome-wide map. It also facilitates access to the T2D-Db candidate gene page and external links to the UCSC genome browser and NCBI Gene. Figure 3 displays the processing of a sample query to obtain a SNP map in a user selected chromosomal region.

g) Protein-protein interaction data can be accessed for each candidate gene through data search as well as interaction map options. Cytoscape, a network visualization and analysis software has been integrated into T2D-Db, which allows the user to view the protein interactors as a network. This option is available for viewing the protein-protein interaction data for each candidate gene. Figure 4 displays protein interactors from APID and PIP database for a candidate gene. The interaction data can be analyzed and functionally elaborated with the help of interaction network in Cytoscape.

h) A complete list of genes involved in T2DM-associated risk factors/complications has been provided in the 'Risk Factors/Complications' section of the database. Search options similar to that of querying candidate gene information have also been provided in this module.

i) The candidate genes and candidate regions have been collected through careful and intensive literature search. Depending on this information all other data subsets have been collected from other external databases as well as from relevant research articles. The statistics for each data subset provides the information of total number of the entries under each category. The statistics page points out the total number of candidate genes (254 genes) and regions (25, specifically for human), their unigene cluster IDs (275), homologene IDs (251), transcript IDs (700), gene ontology terms (1,615), markers like SNPs (54,692) and ESTs (98,727), the expression values obtained from microarray studies (16,946), biochemical pathways (989), tissue-specific expressions (184), interacting proteins (5961) and the research articles (7,302) read and finally used to validate the authenticity of data and related information. It also gives the total number of risk factors (6) and complications (7) associated with type 2 diabetes. This page also facilitates a hyperlink from each entry to the corresponding page, which displays more detailed information. We have an ongoing effort to update the data under each category.

j) T2D-Db has 'save' options in every section of the database. This option is helpful in saving the selected information in a tab-delimited text file. The entire database categorized into different data types, can be download from the 'Downloads' section.

k) A separate section invites data updation from the users. It provides a specific form (tab de-limited text) for each data subset category that is required to be filled to upload the data. T2D-Db team will validate the data and then upload in the database.

## Conclusion

Most of the candidate genes and chromosomal regions have been studied individually and information on genetics, molecular biology and biochemistry of each candidate gene has been reported in the literature. An integrated, curated and up to date resource bringing together all these data is needed to facilitate the research on diabetes. The user friendly web interface of T2D-Db helps users to access the systematically organized data on almost all molecular components reported in the literature. Since it is the first attempt in the public domain to integrate the data on molecular factors involved in T2DM, the database is expected to serve as a unique repository and will be highly useful to the diabetes research community world wide.

The resource will be periodically updated with the further enhanced features and information. We further intend to develop a ranking scheme for the tissue specific expression values so that data from different sources can rationally be presented. The microarray expression module could to be integrated with programs, which can cluster, rank and compare differentially expressed genes across experiments. It is also proposed to implement tools, which can track all the relevant genomic elements in a selected chromosomal region (T2DM candidate region) of an organism. T2D-Db aims to provide the published information of the patients' clinical data.

## Availability and requirements

Project name: T2D-Db.

Project home page: 

Operating system: Platform independent

Programming languages: Java 2, CGI-Perl 5.8.8, MySQL 5.0.22, Apache HTTP server 2.2

Licence: GNU GPL, FreeBSD

For non-academic user: licence needed. Contact to the corresponding author.

## Abbreviations

T2DM: type 2 diabetes mellitus; T2D-Db: type 2 diabetes database; SNP: single nucleotide polymorphism; EST: expressed sequence tag; NIDDM: non-insulin dependant diabetes mellitus; ATP: adenosine triphosphate; NEFA: non-esterified fatty acid; TNF-α: tumor necrosis factor-α; CAPN10: calpain10; PPAR: peroxisome proliferator-activated receptor; PGC1: PPAR-γ coactivator 1; PPARG: Pro12Ala PPAR-γ; KCNJ11: potassium inwardly-rectifying channel, subfamily J, member 11; HNF4α: hepatocyte nuclear factor-4 alpha; GLUT2: glucose transporter 2; TCF7L2: transcription factor 7-like 2 gene; RBP4: retinol binding protein 4; T1D: type 1 diabetes; NCBI: national centre for biotechnology information; OMIM: online mendelian inheritance in man; MGI: mouse genome informatics; RGD: rat genome database; DoTS: database of transcripts; KEGG: kyoto encyclopedia of genes and genomes; APID: agile protein interaction dataAnalyzer; PIP: potential interactions of proteins; GEO: gene expression omnibus; HTML: hypertext markup language; DHTML: dynamic HTML; SQL: structured query language; CGI-Perl: common gateway interface-practical extraction and report language.

## Authors' contributions

SA has designed, developed the entire database concept, validation of the contents, supervised the database development and written the manuscript. ND has given useful and critical suggestions for improving the database, she also has participated in the work design, aseesment and manuscript writing. UK was involved in collecting all the data required for the database, curated with literature evidence and validated. PN majorrly participated in the programming of database development and implementation of software. SSL has contributed in the backend database design and programming. SS and US participated in data validation and manuscript preparation. MN has participated in programming.

## Supplementary Material

Additional file 1 Data flow through T2DDb application. The entire dataflow through eight categories. Three visual maps are used here to display SNP data, microarray gene expression data and EST data corresponding to each candidate gene. Gene information has been obtained from NCBI Gene Entrez database. Click here for file
