# Supplementary material for: T2D-Db: An integrated platform to study the molecular basis of Type 2 diabetes
Source: BMC Genomics. 2008 Jul 7;9:320. doi: 10.1186/1471-2164-9-320 (PMC2491641; doi:10.1186/1471-2164-9-320)
Supplement: Additional file 1 — Data flow through T2DDb application. The entire dataflow through eight categories. Three visual maps are used here to display SNP data, microarray gene expression data and EST data corresponding to each candidate gene. Gene information has been obtained from NCBI Gene Entrez database. [file 1471-2164-9-320-S1.pdf]

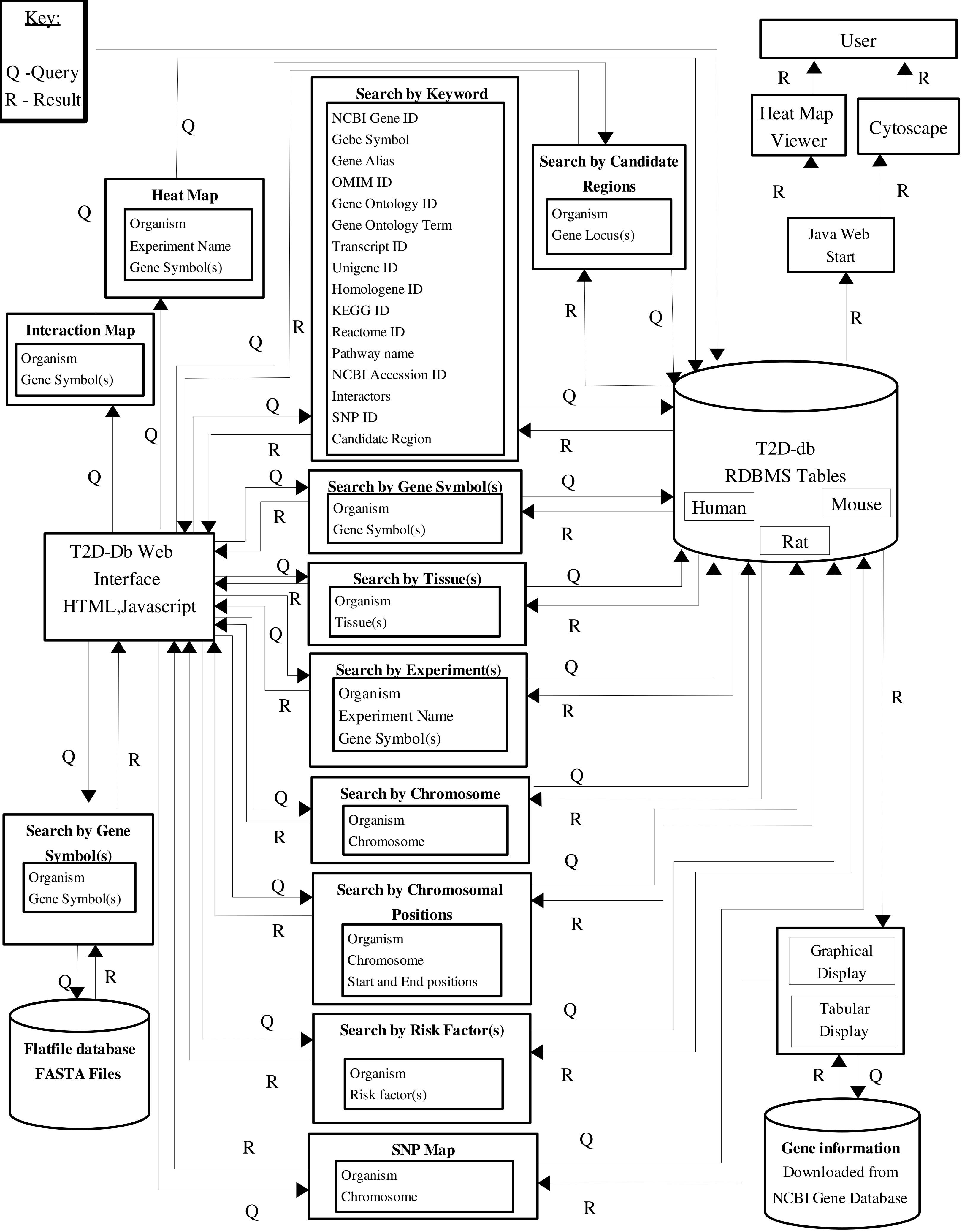
Key:
Q -Query
R - Result
User
Heat Map Viewer
Cytoscape
Java Web Start
Search by Keyword
NCBI Gene ID
Gebe Symbol
Gene Alias
OMIM ID
Gene Ontology ID
Gene Ontology Term
Transcript ID
Unigene ID
Homologene ID
KEGG ID
Reactome ID
Pathway name
NCBI Accession ID
Interactors
SNP ID
Candidate Region
Search by Candidate Regions
Organism
Gene Locus(s)
Heat Map
Organism
Experiment Name
Gene Symbol(s)
Interaction Map
Organism
Gene Symbol(s)
T2D-db
RDBMS Tables
Human
Mouse
Rat
T2D-Db Web Interface
HTML,Javascript
Search by Gene Symbol(s)
Organism
Gene Symbol(s)
Search by Tissue(s)
Organism
Tissue(s)
Search by Experiment(s)
Organism
Experiment Name
Gene Symbol(s)
Search by Chromosome
Organism
Chromosome
Search by Chromosomal Positions
Organism
Chromosome
Start and End positions
Search by Risk Factor(s)
Organism
Risk factor(s)
SNP Map
Organism
Chromosome
Search by Gene Symbol(s)
Organism
Gene Symbol(s)
Flatfile database
FASTA Files
Graphical Display
Tabular Display
Gene information
Downloaded from
NCBI Gene Database

Supplementary Figure 1. Data flow through T2D-Db application

The entire data flows through eight categories. Query from the user passes from the front-end to the back-end through the CGI scripts and vise-versa. SNP Map, Heat Map and Interaction Map are the three maps used for visual display of SNP data, Microarray data and Protein-Protein Interaction data respectively. Java Web Start is used to launch the Heat Map and Cytoscape application. In SNP Map, the genes are displayed along with markers within a specified genomic region range. The Gene information is obtained from the NCBI Gene Entrez Database. A Flat-file database is also maintained in order to store the FASTA sequence files for each gene.
